# Partnership and fertility trajectories of immigrants and descendants in the United Kingdom: A multilevel multistate event history approach

**DOI:** 10.1080/00324728.2022.2144639

**Published:** 2022-11-22

**Authors:** Júlia Mikolai, Hill Kulu

**Affiliations:** University of St Andrews

**Keywords:** fertility, partnerships, multistate event history analysis, immigrants, descendants, United Kingdom

## Abstract

We study the interrelationships between partnership and fertility trajectories of immigrant women and female descendants of immigrants using the UK Household Longitudinal Study. We propose a novel multistate event history approach to analyse the outcomes of unpartnered, cohabiting, and married women. We find that the partnership and fertility behaviours of immigrants and descendants from European and Western countries are similar to those of native women: many cohabit first and then have children and/or marry. Those from countries with conservative family behaviours (e.g. South Asian countries) marry first and then have children. Women from the Caribbean show the weakest link between partnership changes and fertility: some have births outside unions; some form a union and have children thereafter. Family patterns have remained relatively stable across migrant generations and birth cohorts, although marriage is being postponed in all groups. Our findings on immigrants support the socialization hypothesis, whereas those on descendants are in line with the minority subculture hypothesis.

## Introduction

The last decade has witnessed a significant increase in research on immigrant and ethnic minority families in Europe. One stream investigates childbearing patterns among immigrants and descendants of immigrants to understand whether their childbearing behaviour is similar to that of the native population and the reasons for possible differences (e.g. Kulu 2005; Kulu and Hannemann 2016a; Andersson et al. 2017; Baykara-Krumme and Milewski 2017; Kulu et al. 2017). Another stream focuses on the partnership experiences of immigrants and descendants, aiming to determine whether immigrants exhibit partnership patterns similar to those of native women and whether and how partnership patterns differ across migrant generations (Hannemann and Kulu 2015; González-Ferrer et al. 2016; Hannemann et al. 2020). These studies show significant diversity in the childbearing and partnership patterns of immigrants and descendants, and they discuss this heterogeneity in the context of migrant and minority integration and social inequalities.

Previous studies have improved our understanding of the factors that influence the fertility and partnership behaviours of immigrants and descendants. However, partnership transitions and childbearing are inherently interrelated in individuals’ lives and should thus be investigated together. This is critical in the context of increasingly diverse family trajectories, which imply that different partnership trajectories may lead to the same childbearing patterns. Both native and immigrant women used to follow largely conservative family formation pathways—they married first and had children within marriage—but immigrants, especially those from low-income countries, had more children. With increasing family diversity, native and migrant women may experience different partnership and fertility trajectories. For example, one group may marry first and have children within marriage, whereas another group may cohabit first, have a child, marry, and have another child. Yet another group may have children in a union but experience separation thereafter. With increasing family complexity and diversity (Thomson 2014), studying partnership and fertility pathways together is critical to understanding how and why immigrants and descendants may differ from the native population.

In this study, we investigate the interrelationships between partnership and fertility trajectories of immigrants and descendants and compare these to the experiences of those native to the United Kingdom (UK). We focus on women due to the volume and complexity of the results (results for men are available on request). This study has three key novelties. First, by analysing partnership and fertility trajectories together, we shed new light on the partnership context of childbearing among immigrants and descendants. Although a vast literature exists on both life domains separately, our knowledge with respect to the partnership context of childbearing, as well as the different pathways leading to these, remains limited among immigrants and descendants.

Second, most studies on migrant partnerships and fertility have focused on a single event, often first marriage or childbirth. Recent studies have analysed competing partnership transitions, especially transition to first union (cohabitation vs marriage) or to first and higher-order births, often controlling for partnership status (Andersson et al. 2015; Kulu et al. 2017; Hannemann et al. 2020). However, we do not know how entire partnership and fertility trajectories are interrelated in the lives of immigrants and descendants. We propose a novel analytical strategy to model complex partnership and fertility transitions jointly in a multilevel multistate event history framework. This strategy enables us to solve the three main challenges that we face when modelling complex partnership and family formation pathways together: (1) studying several competing outcomes jointly; (2) studying repeated partnership formations, dissolutions, and childbirths; and (3) studying the role of multiple ‘clocks’ (i.e. age, union duration, and/or time since previous birth and/or separation).

Third, we study changes in the link between partnership and fertility among immigrants and descendants. Comparing the intersection of these two life domains across birth cohorts allows us to explore whether and how the link between partnership and fertility transitions has changed among both immigrants and descendants in comparison to native women. This study significantly contributes to our understanding of immigrant and ethnic minority integration over time and across different migrant generations.

We use high-quality longitudinal data from the UK, a country with a long immigration history. Over the last two decades, the share of foreign-born individuals in the UK has grown, from 8 per cent in 2004 to 14 per cent in 2019 (Office for National Statistics 2019). The UK has also experienced migration from many parts of the world, including South Asia, the Caribbean, Africa, and Europe. This makes the UK an interesting case study for analysing the partnership and family formation pathways of immigrants and descendants.

## Background

Five interrelated and sometimes competing hypotheses have been put forward to explain partnership and fertility differences between immigrants and the native population (e.g. Pailhé 2015; Kulu et al. 2019). According to the *socialization* hypothesis, the reason for differences in partnership and fertility between immigrants and the native population is that immigrants’ preferences and behaviours are influenced by the norms and behaviours that were dominant in their childhood environment. By contrast, the *adaptation* (or *assimilation*) hypothesis argues that the host country context has the most influence on their family behaviour. The *selection* hypothesis stipulates that immigrants’ partnership and fertility behaviours are similar to those of the native population because immigrants are a select group: their norms and preferences are different from those in their country of origin and similar to those in the host country. The *disruption* hypothesis suggests that fertility immediately after migration will be low and marriage will be delayed due to the disruptive nature of migration. Over time, fertility and marriage levels will return to normal (Kulu and González-Ferrer 2014; Adserà and Ferrer 2015). Finally, the *interrelation of life events* hypothesis emphasizes that migration and family dynamics are interrelated: many people, especially women, move to form or reunite a family (Andersson 2004; Milewski 2007; Kulu et al. 2019).

Recent studies in Europe have focused increasingly on the partnership and fertility behaviours of the descendants of immigrants (i.e. the second generation). Several hypotheses have been proposed to explain differences in partnership and fertility between the native population and the second generation. As the second generation are born and socialized in the host country but within a family of immigrants (Adserà and Ferrer 2015), some groups are socialized primarily into the norms and behaviours of the native population, whereas others may grow up in a minority subculture displaying norms, preferences, and behaviours that are different from those of the native population (Kulu et al. 2019). This is often referred to as the *minority subculture* hypothesis (Kulu et al. 2019). Additionally, the *minority group status* hypothesis (Milewski 2010a) argues that some groups of descendants face discrimination, which also influences social relationships and thus partnership and fertility decisions (Glick et al. 2006). For example, discrimination against minorities in the labour market may reduce women’s opportunities for social mobility and hence they may decide to enter the ‘motherhood track’ (Kulu et al. 2019). Finally, although the *selection* hypothesis is not per se relevant for the second generation, selection effects from the parents’ generation could be extended by transmission of a preference for higher education and/or employment (Kulu et al. 2019).

### Partnership transitions among immigrants and descendants in Europe

Previous studies on the partnership experiences of immigrants have investigated the timing and type of union formation and dissolution, comparing the experiences of the first and second generation with those of the native population across several industrialized countries. Most studies have focused on marriage and divorce, but some have also explored cohabitation, separation, and repartnering (Andersson et al. 2015; Pailhé 2015; González-Ferrer et al. 2016; Kulu and Hannemann 2016a; Kuhnt and Krapf 2020).

In the UK, some early studies showed that partnership formation among South Asian (Indian, Pakistani, and Bangladeshi) immigrants was characterized by early and universal marriage; cohabitation and separation were very rare (Berrington 1994, 1996). By contrast, Caribbean immigrants (primarily from Jamaica but also from other Caribbean countries) exhibited lower marriage rates and higher cohabitation and separation rates. Among the second generation, partnership patterns converged towards those of the native population (Berrington 1994, 1996). More recently, Hannemann and Kulu (2015) found that direct marriage is still the most prevalent form of union formation, and cohabitation and separation remain rare among immigrants and descendants from India, Pakistan, and Bangladesh. Caribbean immigrants and descendants exhibit high cohabitation, low marriage, and high divorce rates, whereas partnership patterns for immigrants from Europe are similar to those of native British women.

Studies in other European countries have generally found similar trends to those in the UK. First, immigrants from countries with conservative partnership patterns show high marriage rates and low cohabitation and separation rates (Kulu and Hannemann 2016a). These patterns have been observed in Sweden for immigrants and descendants from the Middle East, Iran, Turkey, and Southeast Asia (Andersson et al. 2015); in Germany for Turkish and ethnic German immigrants (Kuhnt and Krapf 2020); and in France for Turkish and North African immigrants (Pailhé 2015). Interestingly, in Sweden, some of these groups exhibited higher rates of divorce and remarriage than native Swedes (Andersson et al. 2015). A recent comparative study across the UK, France, Spain, and Estonia showed that the partnership patterns of immigrants from countries with conservative family patterns are similar across the destination countries (Hannemann et al. 2020).

Second, the partnership formation and dissolution patterns of migrants from sub-Saharan Africa, Latin America, and Europe are more diverse across host countries than those of migrants from countries with conservative family formation patterns (Kulu and Hannemann 2016a). For example in Spain, immigrants from Latin America are more likely than the native Spanish to cohabit and to separate (González-Ferrer et al. 2016), whereas Eastern Europeans are more likely to marry and those from Western and Southern Europe are more likely to separate than the native Spanish (González-Ferrer et al. 2016). In France, risks of direct marriage and cohabitation are lower for immigrant women from sub-Saharan Africa but higher for their male counterparts than among native French women and men, respectively (Pailhé 2015). Descendants of sub-Saharan African migrants are equally likely to marry but less likely to cohabit than their native French counterparts (Pailhé 2015). In Sweden, risks of marriage, divorce, and repartnering are lower for Southern Europeans than native Swedes (Andersson et al. 2015). However, in France, immigrants from Southern Europe display higher direct marriage rates and men also display higher cohabitation rates than the native French (Pailhé 2015). Southern European men exhibit lower direct marriage rates and women lower cohabitation rates than the native French.

### Fertility of immigrants and descendants in Europe

There is a vast literature on the fertility of immigrants and descendants across Europe (Kulu and González-Ferrer 2014; Adserà and Ferrer 2015; Kulu et al. 2019). Most studies have compared the fertility of the first and second generations with that of the native population in the host country, although some have compared the behaviours of immigrants with those of non-immigrants in their origin country (e.g. Baykara-Krumme and Milewski 2017; Puur et al. 2017; Lindström et al. 2022). Although many studies have included partnership status as a control variable, they have not explored the interrelationships between entire partnership and childbearing trajectories.

Previous studies have found significant heterogeneity in the fertility of immigrants and descendants across Europe (Kulu et al. 2019). We highlight the most recent and most relevant findings. In the UK, first-birth risks are higher for Pakistani and Bangladeshi immigrants but lower for European and other immigrants than for native women (Kulu and Hannemann 2016b). Among the second generation, there are few differences in first-birth risks compared with native women. However, fertility levels are still relatively high among Pakistani and Bangladeshi women. By contrast, descendants of Caribbean immigrants exhibit lower second-birth risks and similar third-birth risks compared with native UK women. The high fertility among women of Pakistani and Bangladeshi descent was attributed to cultural factors, as the patterns persisted even after controlling for education and employment (Kulu and Hannemann 2016b). Wilson and Kuha (2018) found that fertility levels among immigrants’ descendants were more similar to those of native women if they grew up in an area which was less residentially segregated. Residential segregation explained some of the high fertility among women of Pakistani and Bangladeshi descent, providing indirect evidence for the importance of cultural factors.

Similar patterns to the UK have been found across Europe. First, the fertility levels of non-Western immigrants are higher than those of native populations (Kulu et al. 2017). For example, groups with higher first birth rates than their native counterparts (even after controlling for educational differences in some cases) include Turkish and sub-Saharan African immigrants in France; Turkish, Moroccan, and Italian immigrants in Belgium; Turkish immigrants in Germany; immigrants from the Maghreb and Latin America in Spain; and Albanian, Moroccan, and Romanian immigrants in Italy (Milewski 2007, 2010b; Mussino and Strozza 2012a, 2012b; González-Ferrer et al. 2017; Kulu et al. 2017; Pailhé 2017; Van Landschoot et al. 2017). The risks of a second and third birth are relatively high in France among immigrants from the Maghreb and Turkey, in Belgium among those from Morocco and Turkey (Kulu et al. 2017), and in Spain among those from the Maghreb (González-Ferrer et al. 2017). Interestingly, immigrant women (except those from high-income countries) in Spain display lower second-birth rates than native Spanish women (González-Ferrer et al. 2017).

Second, although fertility differences between the descendants of immigrants and native women tend to be smaller than those between immigrant and native women, significant differences persist in most European countries. Among the second generation in Europe, first-birth rates are either similar to or somewhat lower than those of native women (Kulu et al. 2017). In Sweden, descendants of immigrants from most origin groups show depressed first- and second-birth rates compared with native Swedes; however, the risk of a third birth is high among many groups (Andersson et al. 2017). The patterns are somewhat different in Switzerland: first-birth risks are higher among immigrants than native women, whereas second-birth risks are lower among both immigrants and descendants from all origin countries (Rojas et al. 2018).

Finally, there are fewer differences between the fertility levels of native populations and immigrants/descendants from other European and Western countries. The descendants of European immigrants in Sweden exhibit similar or lower first-birth rates to native Swedish women (Scott and Stanfors 2011). In Germany, fertility levels are similar for immigrants from Southern Europe and native Germans (Milewski 2007, 2010b), whereas in Belgium, second- and third-birth rates are lower for second-generation Southern European women than native women (Van Landschoot et al. 2017).

To summarize, existing evidence on why immigrants’ partnership and fertility behaviours differ from those of native women is mixed. Immigrants from European and Western countries show similar partnership and fertility patterns to the native population. This might be either because partnership and fertility patterns are similar in their country of origin, supporting the socialization hypothesis, or because many of them are married to native partners (who influence their partnership and fertility behaviours), supporting the adaptation hypothesis. Immigrants from countries with conservative family patterns (e.g. Turkey, South Asian, and North African countries) show higher fertility, higher marriage rates, and lower rates of cohabitation and separation than the native population, supporting the socialization hypothesis. The evidence is less clear when studying the family behaviours of immigrants from other non-European regions (e.g. Africa, Latin America, and the Caribbean).

Regarding the partnership and fertility behaviours of the second generation, the descendants of European immigrants tend to exhibit similar family behaviours to the native population. By contrast, patterns of union formation and fertility among the second generation from Turkey, South Asia, and North Africa are more similar to those of the first generation than to native women, supporting the minority subculture hypothesis. At the same time, divorce levels in the second generation are in between those of the first generation and native women (Pailhé 2015; Hannemann et al. 2020), providing partial support for the adaptation hypothesis.

## The context of the UK and the main immigration countries

The post–Second World War economic recovery and growth during the 1950s and 1960s attracted immigrants from the New Commonwealth, including Caribbean countries, India, Pakistan, and Bangladesh (Dale and Ahmed 2011; Dubuc 2012). Due to the introduction in 1962 of severe restrictions on entry to Britain from the Asian subcontinent, many initially temporary immigrants decided to settle in the UK permanently and bring their families (Dale and Ahmed 2011). Thus, the migration flows of the 1960s were characterized by family reunification (Coleman and Dubuc 2010; Dale and Ahmed 2011; Dubuc 2012). In the 1970s, immigration from Caribbean countries started to decline, whereas immigration from sub-Saharan Africa increased (Coleman and Dubuc 2010; Dubuc 2012). More recently, many migrants have arrived from China and from countries recently joining the European Union, especially Poland (Waller et al. 2014; Robards and Berrington 2016).

The share of ethnic minorities has also increased considerably over time. In the 1991 Census, 7 per cent of the UK population declared a non-White ethnicity. The largest groups identified themselves as being of Indian, Caribbean, Pakistani, Chinese, or Bangladeshi origin. By 2011, the share of ethnic minorities had increased to 20 per cent. In 2020, the most common non-UK countries of birth were India, Poland, Pakistan, the Republic of Ireland, and Romania (Office for National Statistics 2020).

Partnership formation and dissolution patterns, as well as fertility levels and timing, have changed remarkably across high-income countries, including the UK, over the last few decades. This implies that the interrelationships between partnership and fertility have also changed. For example, an increasing share of first unions start as cohabitations (Ermisch and Francesconi 2000), more children are born to cohabiting parents (Perelli-Harris et al. 2010), and divorce and repartnering rates have increased, leading to the emergence of multi-partner fertility and complex families (Thomson et al. 2012; Thomson 2014).

Family behaviours in many immigrant origin countries differ considerably from those in the UK (Hannemann and Kulu 2015). For example, South Asian countries (i.e. India, Pakistan, and Bangladesh) were traditionally characterized by early and universal marriage, high fertility, and low levels of non-marital childbearing (Alexander et al. 2006). Between 1970 and 2019, total fertility declined remarkably in India (from 5.6 to 2.1), Pakistan (from 6.8 to 3.6), and Bangladesh (from 6.9 to 2.0) (United Nations 2019a). At the same time, the singulate mean age at marriage (SMAM) has increased by around three years for both men and women (United Nations 2019b). However, it remains considerably lower compared with the UK’s, especially for women (21 in India, 23 in Pakistan, 19 in Bangladesh, and 27 in the UK) (United Nations 2019b).

These partnership and family formation patterns are a consequence of the family system and the customs, norms, and values surrounding partnership selection, marriage, and childbearing (Yeung et al. 2018). In South Asia, extended or multigenerational households are seen as the ideal family type, and relationships within households are shaped by hierarchies of sex, age, and status (Shaw 2004). Marriages are arranged at young ages by parents or other relatives, with the aim of preserving or enhancing the family’s status (Shaw 2004; Yeung et al. 2018), although increased levels of education have led to increasing involvement of brides in partner selection (Yeung et al. 2018). Childbearing occurs in marriage not only because of the young age at marriage but also because non-marital fertility is not deemed acceptable (Yeung et al. 2018).

There are considerable heterogeneities within South Asian countries, for example by religion (Yeung et al. 2018). Bangladesh and Pakistan are predominantly Muslim countries, whereas India is dominated by the Hindu religion (around 14 per cent are Muslim) (Yeung et al. 2018). The few studies that have explored the role of religious differences in fertility within South Asian countries showed that Muslim wives had more children and were more likely to desire additional children than non-Muslim wives (Morgan et al. 2002; Heaton 2011).

By contrast, Caribbean countries are characterized by the presence of both conjugal and multigenerational households, often headed by a senior woman (Shaw 2004). Young adults may marry their romantic partner, live in a ‘common-law’ relationship, or form a so-called ‘visiting relationship’ (i.e. co-reside with their parents while at the same time forming a relationship outside the household) (Miner 2003; Shaw 2004). Children born from visiting relationships typically live with their mother, and grandmothers play a more prominent role in childrearing than fathers (Berrington 1996; Shaw 2004). Caribbean countries report low marriage rates and high repartnering rates, and childbearing often precedes union formation (Berrington 1994; Miner 2003; Shaw 2004). In the Caribbean, total fertility declined from 4.7 in 1970 to 2.1 in 2019 and the SMAM for Jamaican women (the most common Caribbean origin country in the UK) increased from 21 to 25 between 1970 and 2011 (for men it was 33 in the 1970s and 35 in 1991; no information is available for later years) (United Nations 2019a, 2019b).

There is also considerable variation in partnership and fertility behaviours across European countries. For example, whereas Northern and Western European countries have often been the forerunners of new demographic behaviours, Southern European countries exhibit low divorce rates, high marriage rates, and low fertility (Billari and Kohler 2004). However, partnership and fertility patterns across Europe are more similar overall to the patterns in the UK than to those in many non-European countries (Hannemann and Kulu 2015).

## Expectations

Based on the literature, we derive five hypotheses. First, among immigrants and descendants from culturally similar countries (European and Western countries), we expect that the links between partnership and fertility will be similar to those among the native UK population (*similarity* hypothesis). We expect similar propensities to have children while unpartnered and within cohabitation or marriage, as well as similar levels of cohabitation, marriage (both direct marriage and marriage preceded by cohabitation), and separation.

Second, among immigrants and descendants from countries with conservative family behaviours (South Asian countries), we expect that the links between partnership and fertility will be stronger than among native women (*stronger links* hypothesis). This means very little cohabitation, non-marital childbearing, and separation, as well as higher levels of fertility within marriage than among native women.

Third, among immigrants and descendants from the Caribbean, we expect that the links between partnership and fertility will be weaker than among native women (*weaker links* hypothesis). This implies higher levels of non-marital childbearing, cohabitation, and separation than among native women.

Fourth, when comparing the family and fertility behaviours of the first and second generations, we expect to observe a convergence to the behaviour of the native population; that is, we expect that the strength of the link between the partnership and fertility behaviours of second-generation immigrants will be in between that of native and immigrant women. We expect this to be especially the case for immigrants from culturally similar (European and Western) countries and to a smaller extent among those from culturally dissimilar (South Asian) countries (*convergence* hypothesis).

Finally, the link between partnership and fertility histories is expected to be weaker among more recent than earlier birth cohorts. More recent birth cohorts of all origin groups are expected to be more likely to experience cohabitation, separation, and childbearing within cohabitation. We expect to observe some changes across birth cohorts in all migrant groups and generations, although we expect this especially among the second generation (*cohort change* hypothesis).

## Data and sample

We use data from Waves 1–9 (2009–19) of the UK Household Longitudinal Study (UKHLS) (University of Essex 2020b), a large, nationally representative household panel. All adult household members are interviewed annually about topics including partnerships, fertility, employment, education, income, housing, and health. The UKHLS contains retrospective partnership and fertility histories including the start and end dates (year and month) of unions and the dates (year and month) of births. Additionally, the panel waves provide prospective information on changes in partnership status and the birth of (additional) children since last interview (Nandi et al. 2020). We use the Marital and Cohabitation Histories file (University of Essex 2020a). Additional analysis (available on request) comparing data from the UKHLS and the Office for National Statistics (ONS) revealed that the quality of both partnership and fertility histories in the UKHLS is very high: our weighted estimates from the UKHLS were comparable to and consistent with those provided by ONS. We detected some inconsistencies for the most recent cohorts, which we account for by censoring observations in these cohorts at younger ages (see Methods and analytical strategy section).

The UKHLS provides an exceptional opportunity to study the lives of immigrants and descendants from different origin countries. It contains two boost samples, where ethnic minorities from high ethnic minority concentration areas were oversampled to ensure a sufficiently large sample size (McFall et al. 2019).

The UKHLS has collected information on about 30,000 households, corresponding to around 51,000 individuals. Our analytical sample is restricted to women born after 1940, who were at least 16 years old at the time of the interview, were original or permanent sample members, and completed a full interview in Wave 1 or were included in the boost samples. We remove women whose first birth or first union occurred before age 16 (549 cases) and those with missing information on the year of birth of their first child (228 cases) or both their mother’s and father’s country of origin (29 cases). The resulting sample consists of 27,943 women.

## Methods and analytical strategy

**Figure 1:**
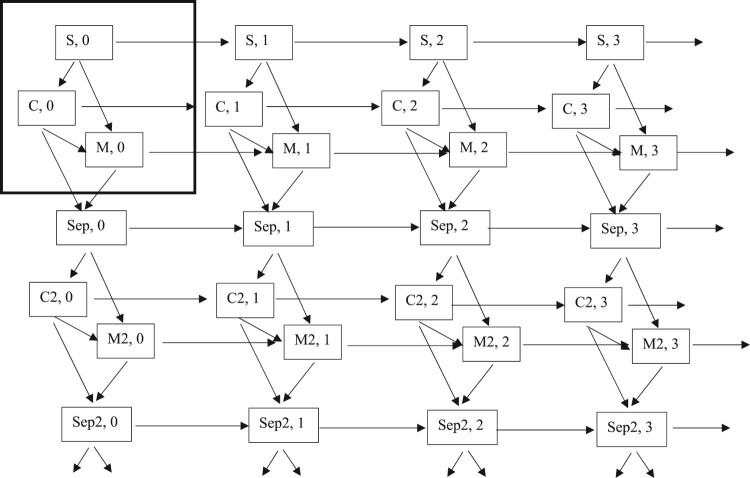
Schematic representation of repeated partnership and fertility transitions *Notes*: S = never partnered; C = cohabitation; M = marriage; Sep = separation; C2 = second union (cohabitation); M2 = second union (marriage); Sep2 = second separation; the numbers 0–3 represent women’s parity (i.e. no children, one child, two children, and three children). The large box in bold in the top left indicates the partnership and fertility transitions we study as repeated events. Moving to the right from this box, we depict transitions to higher-order births, whereas moving downwards, we depict transitions to higher-order unions. Individuals can experience more than two unions/separations and more than three births.

To study changes in partnership status and parity simultaneously, we estimate multistate event history models. These models are an extension of conventional event history models: rather than analysing a single transition, individuals move between different states as they age (Putter et al. 2007; Mikolai et al. 2018). Figure 1 shows the states (boxes) and the possible transitions (arrows) between them. Each box shows a combination of partnership status and parity. For example, the state ‘C2, 3’ represents individuals who are in a second union, which is a cohabitation, and who have three children.

We observe individuals from age 16 when they are never partnered (i.e. single) and childless (see large box in bold in Figure 1). Single and childless (S, 0) individuals can either cohabit (S, 0 → C, 0), marry directly (S, 0 → M, 0), or have a first child (S, 0 → S, 1). Once cohabiting, individuals can either marry (C, 0 → M, 0), separate (C, 0 → Sep, 0), or have a first child (C, 0 → C, 1). Finally, married individuals can either separate (M, 0 → Sep, 0) or have a first child (M, 0 → M, 1). These transitions can be repeated; for example, women can have additional children and separated women can form a new union. Moving to the right from the large box in bold in the upper left corner in Figure 1, we depict transitions to higher-order births, whereas moving downwards, we depict transitions to higher-order unions. Although further transitions are not depicted in Figure 1, individuals can experience more than two unions/separations and more than three births.

As individuals can experience many transitions, we face three challenges: (1) how to model several types of partnership and fertility outcomes; (2) how to account for repeated partnership and fertility transitions; and (3) how to include the roles of both age and time since previous partnership and/or fertility transition. Next, we outline how our innovative modelling strategy solves these challenges.

First, to study the risk of several types of partnership and fertility outcomes, we estimate three sets of competing risks models, for the outcomes of: (1) never partnered; (2) cohabiting; and (3) married women. These models are specified as follows:

(1) $$\ln\;\mu_{i}^{k}(t)=\;\ln\mu_{0}(t)+\underset{j}{\sum }\alpha_{j}x_{ij}+\underset{l}{\sum }\beta_{l}w_{il}(t)+\gamma_{k}z_{i}$$

where $\mu_{i}^{k}$ is the risk of experiencing a transition of type k for individual i. Among single women, this refers to the risk of experiencing a cohabitation, marriage, or childbirth; among cohabiting women, it refers to the risk of marriage, separation, or childbirth; and among married women, it denotes the risk of separation or childbirth. The baseline log-hazard, $\ln\mu_{0}(t)$, is specified as piecewise constant; $x_{ij}$ represents time-constant and $w_{il}$ time-varying variables. We model competing outcomes simultaneously using an extended data set where each individual has k records, corresponding to the number of competing transitions in each set of competing risks models (Cleves et al. 2016). Migrant origin is denoted by $z_{i}$, and γ is a transition-specific parameter to measure its effect. The model assumes a common baseline (or age pattern) for all partnership and fertility transitions, but the risk of each transition can vary by migrant origin. This allows us to estimate the risk of each partnership/fertility transition for individuals from different migrant origins. For all transitions, the baseline is age in months.

Second, individuals can experience these competing partnership and fertility transitions several times. To model repeated transitions, it is possible to estimate separate models for each set of transitions of each order. However, this would lead to many models and inefficient estimates for higher-order unions and births due to small risk populations and small numbers of events. Instead, we extend the multistate model to a multilevel set-up to analyse repeated partnership and fertility transitions. The model is specified as:

(2) $$\ln\;\mu_{im}^{k}(t)=\;\ln\mu_{0}(t)+\underset{j}{\sum }\alpha_{j}x_{ijm}+\underset{l}{\sum }\beta_{l}w_{ilm}(t)+\gamma_{k}z_{im}+\varepsilon_{i}$$

where $\mu_{im}^{k}$ is the risk of experiencing a transition of type k of order m (first or higher-order) for individual i, and $\varepsilon_{i}$ is the individual-level random effect to adjust for the nested structure of the data (transitions are nested within individuals).

Third, most applications of multistate models in demography use only one clock (usually age). But for studying life events such as separation or second and subsequent births and unions, union duration or time since previous event (i.e. separation or birth) are critical in addition to individuals’ age. We use individuals’ age in months as the baseline, but we also account for other clocks: for transitions out of cohabitation and marriage, we include cohabitation or marriage duration, respectively, and for second and higher-order union/parity transitions, we include time since separation or previous birth. The model then becomes:

(3) $$\ln\;\mu_{im}^{k}(t)=\;\ln\mu_{0}(t)+\underset{j}{\sum }\alpha_{j}x_{ijm}+\underset{l}{\sum }\beta_{l}w_{ilm}(t)+\;\delta u_{im}(t)+\tau y_{im}(t)+\gamma_{k}z_{im}+\varepsilon_{i}$$

where $u_{im}(t)$ denotes a time-varying variable of union order, which also includes categories to measure time since separation or union duration, and $y_{im}$ denotes an equivalent variable for birth order and time since previous birth. We simplify the proposed multilevel multistate model: instead of including an individual-level random effect, we correct standard errors of the parameter estimates to account for the nested structure of the data.

Individuals are observed from age 16 until age 50, widowhood, a twin birth, or the time of their last interview. Given some data quality issues for the most recent cohorts, individuals born in 1980–84 are censored at age 30, those born in 1985–89 at age 25, and those born in or after 1990 at age 20.

First, we estimate the three sets of competing risks models (for unpartnered, cohabiting, and married women) with age, birth cohort, migrant origin, level of education, and the relevant duration and union/birth order variables. Second, we estimate three-way interaction effects between type of transition, migrant origin, and birth cohort to study whether and how the link between partnership and fertility has changed across birth cohorts among the first and second generation.

As the UKHLS has a complex sampling design and the minority boost samples come from areas with high ethnic minority concentration, it is important to use weights (McFall et al. 2019). However, the use of cross-sectional or longitudinal weights is not possible in event history analyses where retrospective and prospective information is combined. Additionally, it is not currently possible to incorporate clustered standard errors at both the individual level and the level of the primary sampling unit (i.e. area). For all these reasons, we present unweighted results. However, using the first available cross-sectional weight for each individual (not shown) did not change the results and conclusions of the study.

## Variables

The migrant origin of each individual is determined using information on both their own and their mother’s country of birth. If own country of birth is missing (59 cases), we impute it using self-reported ethnicity. If the mother is UK born (1,693 cases) or her country of birth is missing (17 cases), we use the father’s country of birth. If the respondent is UK born and information on the country of birth is missing for both parents (57 cases) or available only for one UK-born parent (1,156 cases), we use information on individuals’ own ethnicity. Native women are defined as those born in the UK to UK-born parents. Immigrants are defined as those born outside the UK. Descendants of immigrants (i.e. the second generation) were born in the UK but at least one of their parents was born outside the UK. We also distinguish between different origin groups. We compare the experiences of groups from Europe and other Western nations (Australia, New Zealand, Canada, the United States), India, Pakistan, Bangladesh, the Caribbean (primarily Jamaica but also other Caribbean countries), African countries, and other countries (including China and Sri Lanka, among others).

Age is the baseline and is categorized in the following age groups: 16–19, 20–24 (reference), 25–29, 30–34, 35–39, 40–44, and 45–49. We adjust the analysis for union order and duration as well as birth order and duration. The specification of these variables varies across the three competing risks models. For the outcomes of unpartnered individuals, we control for time since previous separation (no separation = reference, 0–1 year, 1–3 years, 3–5 years, and 5+ years) and the order of separation (separated once = reference vs twice or more). To model the outcomes of cohabitations and marriages, we control for cohabitation and marriage duration, respectively (0–1 year = reference, 1–3 years, 3–5 years, and 5+ years), as well as union order (first = reference vs second or higher-order union). All three competing risks models are also adjusted for time since previous birth (no birth = reference, 0–1 year, 1–3 years, 3–5 years, and 5+ years) and birth order (less than two = reference vs two or more children).

We control for several other factors. Birth cohort is divided into three groups (1940–59, 1960–79, and 1980–2003). Level of education is a time-varying variable measured as high (university degree or other higher degrees (e.g. diploma in higher education; teaching or nursing qualification)), medium (A levels), or low (less than completed A levels). Using information on the ages at which respondents left school and left full-time education, we calculate the time at which individuals’ level of education changed. If this information is not available, we impute the ages at completion of medium and high levels of education as ages 18 and 21, respectively, following Kulu and Hannemann (2016b).

## Results

### Descriptive results

Table 1 shows the numbers and proportions of person-months and of partnership and fertility events by migrant origin (Table A1 in the supplementary material shows these statistics by category for all other variables). Native women contributed the largest share of person-months and the largest number of events across all three sets of competing risks models. Nonetheless, there are sufficient numbers of events within each migrant group to conduct detailed analyses on their partnership and fertility transitions. Among unpartnered Bangladeshi women, very few experienced cohabitation and, consequently, even fewer experienced any of the three cohabitation outcomes. To ensure that there are a sufficient number of events in all groups, we merge women of Pakistani and Bangladeshi origin into one group when analysing the outcomes of cohabiting women.

**Table 1:** Number and proportion of person-months, and partnership and fertility events by migrant origin and generation, women (born 1940–2003) in the UK

*Unpartnered women*			*Outcomes*					
	Person-months		Cohabitation		Marriage		Birth	
	*N*	%	*N*	%	*N*	%	*N*	%
*Native women*	2,008,893	61.26	11,581	75.49	7,395	60.21	3,512	57.65
*Immigrants*								
Europe and Western countries	135,508	4.13	709	4.62	401	3.26	158	2.59
India	74,967	2.29	49	0.32	584	4.75	74	1.21
Pakistan	64,905	2.98	18	0.12	580	4.72	104	1.71
Bangladesh	32,842	1.00	5	0.03	396	3.22	57	0.94
Caribbean countries	43,938	1.34	119	0.78	78	0.64	214	3.51
African countries	103,129	3.14	222	1.45	366	3.98	271	4.45
Other countries	338,400	10.32	987	6.43	1,176	9.57	762	12.51
*Descendants of immigrants*								
Europe and Western countries	121,874	3.72	635	4.14	354	2.88	214	3.51
India	54,549	1.66	105	0.68	271	2.21	54	0.89
Pakistan	47,891	1.46	28	0.18	302	2.46	75	1.23
Bangladesh	24,815	0.76	10	0.07	74	0.60	19	0.31
Caribbean countries	74,788	2.28	282	1.84	63	0.51	270	4.43
African countries	40,351	1.23	119	0.78	77	0.63	65	1.07
Other countries	112,458	3.43	473	3.08	166	1.35	243	3.99
*Total*	3,279,306	100	15,342	100	12,283	100	6,092	100
*Cohabiting women*			*Outcomes*					
	Person-months		Marriage		Separation		Birth	
	*N*	%	*N*	%	*N*	%	*N*	%
*Native women*	553,634	76.80	5,810	77.22	4,054	72.85	4,221	77.56
*Immigrants*								
Europe and Western countries	28,262	3.92	330	4.39	278	5.00	136	2.50
India	1,536	0.21	28	0.37	12	0.22	14	0.26
Pakistan	693	0.10	8	0.11	<5	0.07	<5	0.07
Bangladesh	152	0.02	<5	0.05	<5	0.00	<5	0.02
Caribbean countries	5,486	0.76	63	0.84	41	0.74	67	1.23
African countries	8,963	1.24	109	1.45	77	1.38	97	1.78
Other countries	39,467	5.47	474	6.30	357	6.42	297	5.46
*Descendants of immigrants*								
Europe and Western countries	31,859	4.42	311	4.13	221	3.97	220	4.04
India	4,581	0.64	47	0.62	53	0.95	23	0.42
Pakistan	1,165	0.16	12	0.16	16	0.29	13	0.24
Bangladesh	250	0.03	8	0.11	<5	0.04	<5	0.04
Caribbean countries	16,214	2.25	87	1.16	167	3.00	169	3.11
African countries	5,068	0.70	44	0.58	62	1.11	35	0.64
Other countries	23,529	3.26	189	2.51	221	3.97	143	2.63
*Total*	720,859	100	7,524	100	5,565	100	5,442	100
*Married women*			*Outcomes*					
	Person-months		Separation		Birth			
	*N*	%	*N*	%	*N*	%		
*Native women*	2,347,500	69.08	4,042	74.96	18,453	63.04		
*Immigrants*								
Europe and Western countries	110,306	3.25	143	2.65	982	3.35		
India	117,030	3.44	54	1.00	1,051	3.59		
Pakistan	111,493	3.28	75	1.39	1,490	5.09		
Bangladesh	76,021	2.24	40	0.74	1,034	3.53		
Caribbean countries	22,213	0.65	53	0.98	157	0.54		
African countries	79,238	2.33	101	1.87	746	2.55		
Other countries	245,888	7.24	334	6.19	2,492	8.51		
*Descendants of immigrants*								
Europe and Western countries	110,336	3.25	231	4.28	902	3.08		
India	52,934	1.56	49	0.91	544	1.86		
Pakistan	35,600	1.05	73	1.35	528	1.80		
Bangladesh	9,129	0.27	11	0.20	133	0.45		
Caribbean countries	17,541	0.52	51	0.95	138	0.47		
African countries	14,907	0.44	28	0.52	157	0.54		
Other countries	48,123	1.42	107	1.98	463	1.58		
*Total*	3,398,258	100	5,392	100	29,270	100		

*Notes*: Unpartnered women refers to never partnered and separated women. Following Office for National Statistics guidelines for statistical disclosure, we do not disclose the number of events where this is less than five.

*Source*: Authors’ calculations based on data from the UK Household Longitudinal Study (UKHLS), 2009–19.

### Outcomes for unpartnered, cohabiting, and married women

**Figure 2:**
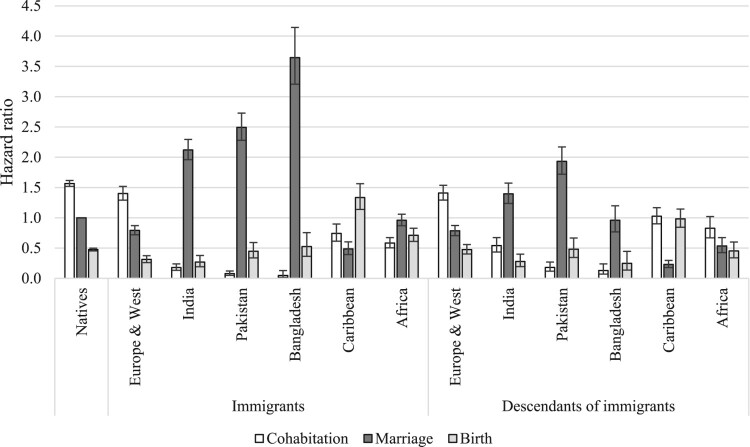
Outcomes for unpartnered women: relative risks of cohabitation, marriage, and childbirth in the UK by migrant origin and generation *Notes*: Unpartnered women refers to never partnered and separated women. Whiskers indicate 95 per cent confidence intervals compared with the reference category (the risk of native women marrying). Results of the full model are shown in Table A2, supplementary material. *Source*: Authors’ calculations based on data from the UK Household Longitudinal Study (UKHLS), 2009–19.

**Figure 3:**
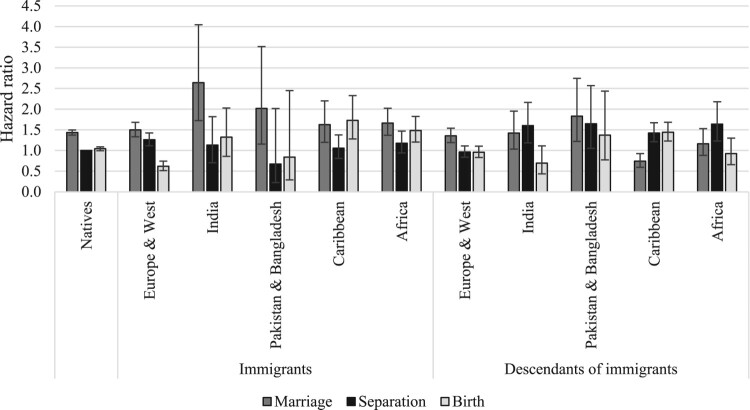
Outcomes for cohabiting women: relative risks of marriage, separation, and childbirth in the UK by migrant origin and generation *Notes*: Whiskers indicate 95 per cent confidence intervals compared with the reference category (the risk of native women separating). Results of the full model are shown in Table A3, supplementary material. *Source*: As for Figure 2.

**Figure 4:**
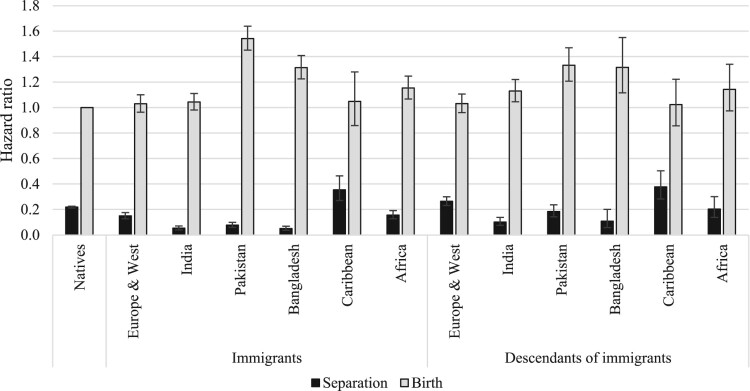
Outcomes for married women: relative risks of separation and childbirth in the UK by migrant origin and generation *Notes*: Whiskers indicate 95 per cent confidence intervals compared with the reference category (the risk of native women having a child). Results of the full model are shown in Table A4, supplementary material. *Source*: As for Figure 2.

Figures 2–4 show the relative risks of the competing events among unpartnered, cohabiting, and married women, respectively. We present interaction effects between migrant origin and the type of transition. We do not present results for the ‘Other’ category to ease readability. The full models are shown in the supplementary material (Tables A2–A4).

Figure 2 shows the relative risks of cohabitation, marriage, and childbearing among unpartnered women by migrant origin. The reference category is the hazard of marrying for unpartnered native women; all other hazards are compared with this. Among unpartnered native women, the risk of cohabitation was the highest, followed by the risks of marriage and childbirth. We find similar patterns among women from Europe and Western countries (for both immigrants and descendants). Among South Asian immigrants (India, Pakistan, Bangladesh), a different pattern emerges: women in this group predominantly married, with much lower risks of childbirth and cohabitation. Cohabitation was rare among these women. Interestingly, these patterns also hold among the descendants of immigrants from South Asia. However, we note two differences. First, among second-generation South Asians, the risk of marriage has declined compared with that of the first generation. Second, among second-generation Indian women the risk of cohabitation was higher than for the first generation, suggesting that some changes have occurred across migrant generations. Unpartnered immigrants from the Caribbean region were most likely to have children, followed by the risks of cohabitation and marriage. Among the second generation, the risk of cohabitation was as high as that of childbirth, whereas the risk of marriage was low. The patterns in terms of most likely outcome changed most among unpartnered African women across migrant generations. Among immigrants, marriage was the most common outcome, whereas among the descendants, cohabitation has become the most prevalent.

The outcomes from cohabitation (marriage, separation, or childbirth) by migrant origin are shown in Figure 3. The reference category is separation risks for native cohabiting women. Among this group and those from Europe and Western countries, marriage was the most likely outcome, with lower risks of childbirth and separation. It is difficult to detect clear patterns among other origin groups due to small numbers, but among South Asian immigrants, marriage seems to have been the most common outcome followed by lower risks of childbirth and separation. Caribbean and African cohabiting immigrants tended either to marry or have a child; their risk of separation was the lowest. In contrast, second-generation Caribbean and African women tended to separate, with lower risks of marriage and birth (those from the Caribbean were equally likely to separate or have a child).

Finally, Figure 4 shows the relative risks of separation and childbirth among married women by migrant origin and generation. The reference category is the risk of having a(nother) child among native married women. The risk of childbirth was considerably larger than the risk of separation among married women in all migrant groups. However, there were some interesting differences in the magnitudes of separation and birth risks across migrant groups. Married Pakistani and Bangladeshi immigrants were more likely to have a(nother) child than those from all other groups; this reflects their higher fertility levels and larger completed family sizes. The birth risks of immigrants from African countries were also somewhat higher than those of native women and European/Western immigrants. However, the birth risks of second-generation African women were comparable to those of other second-generation groups. There were also differences in separation risks: they were the lowest among South Asian immigrants and descendants and highest among Caribbean women. Interestingly, separation risks among second-generation South Asian women were higher than among immigrants.

### Change across birth cohorts

Next, we study whether and how the partnership and fertility interrelationships of immigrants and descendants have changed across birth cohorts using three-way interactions between type of transition, migrant origin, and birth cohort. To ensure a sufficient number of events across migrant groups and birth cohorts, we group together Indian, Pakistani, and Bangladeshi women (i.e. South Asians).

**Figure 5:**
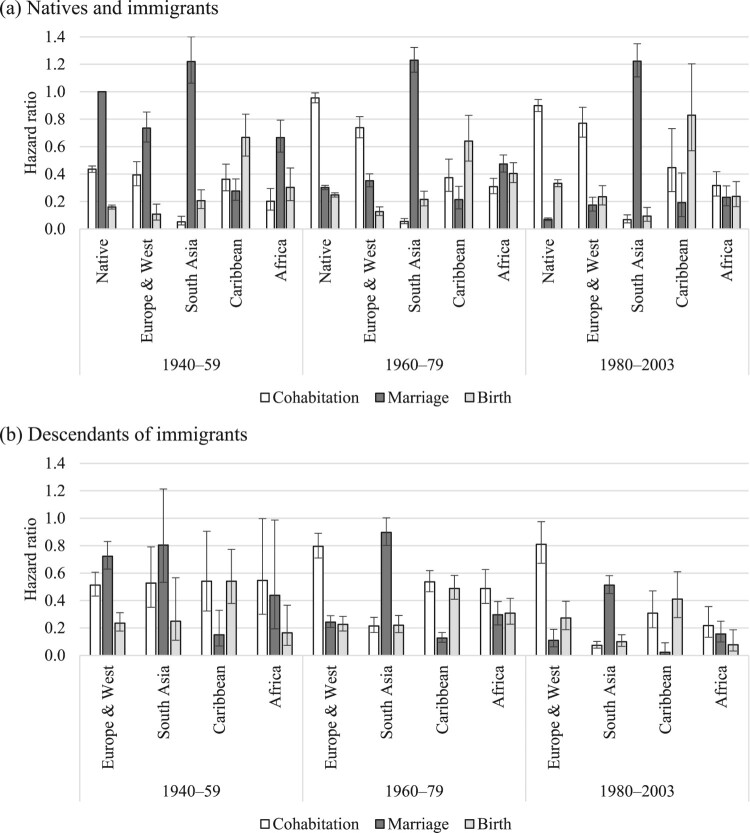
Outcomes for unpartnered women: relative risks of cohabitation, marriage, and childbirth in the UK by migrant origin and birth cohort for (a) native and immigrant women and (b) descendants *Notes*: Unpartnered women refers to never partnered and separated women. Whiskers indicate 95 per cent confidence intervals compared with the reference category (the risk of native women born between 1940 and 1959 marrying). *Source*: As for Figure 2.

Among unpartnered women, the largest change took place among native women. Although among the earliest cohort marriage was the dominant type of first union, among those born in the 1960s and later cohabitation was the primary type of union formation and direct marriage became increasingly rare across birth cohorts (Figure 5). We observe similar patterns among immigrants and descendants from Europe and Western countries. However, we find very little change across birth cohorts among all other immigrant groups. Direct marriage remained the most common outcome among the descendants of South Asian immigrants, although marriage risks declined among the most recent (1980–2003) birth cohort, suggesting the postponement of marriage. Marriage postponement was common among all recent cohorts of descendants.

**Figure 6:**
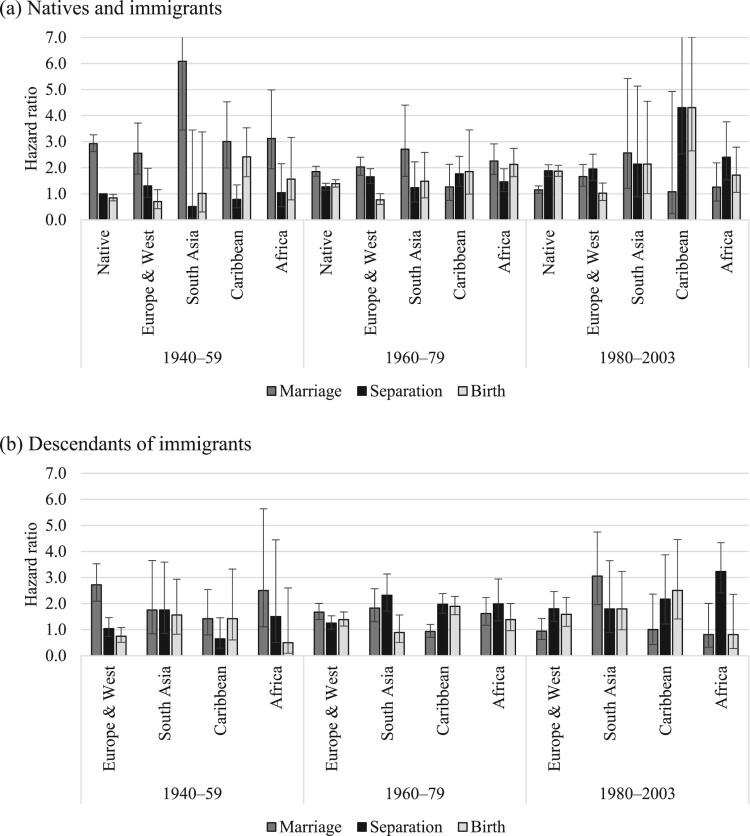
Outcomes for cohabiting women: relative risks of marriage, separation, and childbirth in the UK by migrant origin and birth cohort for (a) native and immigrant women and (b) descendants *Notes*: Whiskers indicate 95 per cent confidence intervals compared with the reference category (the risk of native women born between 1940 and 1959 separating). *Source*: As for Figure 2.

Among native cohabiting women, marriage used to be the most likely outcome, but marriage risks have declined over time and separation risks have increased (Figure 6). Among the most recent cohort, separation and birth were equally likely outcomes of cohabitation, and marriage was the least likely. Among cohabiting immigrants and descendants from European and Western countries, the risk of marriage has also declined over time. It is difficult to detect changes over time in the outcomes of cohabitation among women from all other migrant groups due to the limited number of cohabitations that occurred in the first place.

**Figure 7:**
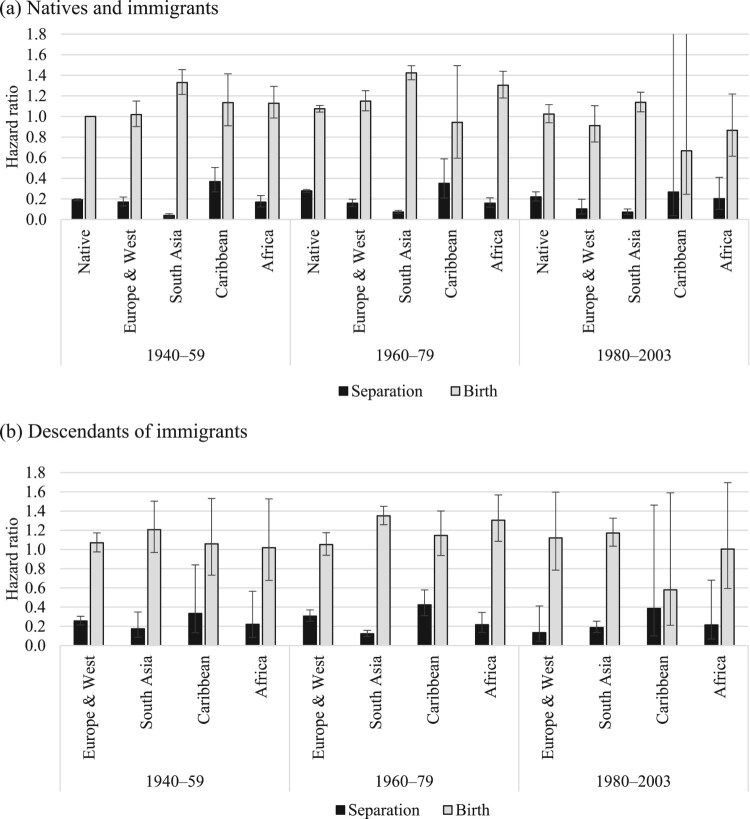
Outcomes for married women: relative risks of separation and childbirth in the UK by migrant origin and birth cohort for (a) native and immigrant women and (b) descendants *Notes*: Whiskers indicate 95 per cent confidence intervals compared with the reference category (the risk of native women born between 1940 and 1959 having a child). *Source*: As for Figure 2.

Among married women, patterns of separation and birth remained stable across birth cohorts among native women as well as immigrants and descendants (Figure 7). South Asian immigrants from the most recent birth cohort were less likely to have a child than their counterparts born earlier; this may indicate the postponement of childbearing but also declining family size. At first glance, the fertility of Caribbean and African immigrants and descendants from the most recent birth cohort seems to have been lower than among earlier cohorts, but the sample is too small to draw definite conclusions. We do not detect any other significant changes in the outcomes of marriage across birth cohorts.

## Conclusion and discussion

We investigated the interrelationships between partnership and childbearing trajectories among immigrants and descendants in the UK using longitudinal data and an innovative analytical strategy that allowed us to analyse repeated competing fertility and partnership transitions jointly and to account for the role of multiple clocks. Our analysis has led to new insights into the partnership context of childbearing among immigrants and descendants in the UK.

First, we analysed the outcomes for unpartnered (single or separated) women by migrant origin and generation. Among British and European/Western unpartnered women, cohabitation is the most common outcome, followed by marriage and childbirth. Unpartnered women of South Asian origin predominantly marry, whereas those of Caribbean descent are most likely to have children while unpartnered. This set of analysis has highlighted that single parenthood is the most likely among women from the Caribbean region and least likely among women from India. Furthermore, among native unpartnered women and those from Europe and Western countries, family formation pathways start primarily with cohabitation, whereas among South Asians the main pathway to family formation is via direct marriage.

Second, we studied the outcomes for cohabiting women. Among native and European/Western women who are cohabiting, marriage is the most likely outcome, with lower risks of childbirth and separation. Caribbean and African cohabiting women tend to either marry or have a child, and their risk of separation is lower. Among South Asians, cohabitation is rare; hence it is not possible to detect any patterns. Among the native population, the results confirm previous studies showing that cohabiting women tend to marry primarily, but many have children within cohabitation. The study has brought new evidence on childbearing within cohabitation among immigrants and descendants. Childbearing within cohabitation is more common among women from Caribbean and African countries than among native women, but those from Europe and Western countries exhibit lower birth risks within cohabitation than native women. Cohabitation, and consequently, childbearing within cohabitation is uncommon among women from South Asia.

Third, we investigated the outcomes for married women and found that among native women, as well as immigrants and descendants from all origin groups, childbirth is a far more likely outcome than separation, especially among women from South Asian countries. At the same time, married women from the Caribbean region are significantly more likely to experience marital separation than women in the other origin groups: a finding that holds among both migrant generations. In line with previous studies, we have shown that fertility is higher among women from Pakistan and Bangladesh than women from other countries, although these differences are smaller among immigrants’ descendants.

These findings are largely in line with our expectations. The strength of the link between the partnership and fertility behaviours of immigrants and descendants from geographically close (and culturally similar) countries (i.e. Europe and Western countries) is comparable to that of native women (similarity hypothesis), whereas women from countries with conservative family behaviours (i.e. South Asian countries) display a close link between partnership and family transitions (stronger links hypothesis). This link is the weakest among Caribbean women (weaker links hypothesis). Contrary to our expectations, we did not find overwhelming evidence for significant changes across migrant generations. We expected that the patterns of the second generation would be in between those of native and immigrant women (convergence hypothesis). We did find some evidence for convergence but only among unpartnered women. For example, second-generation South Asian women are less likely to marry than South Asian immigrants. However, this indicates only slight changes in the partnership behaviours of South Asians, namely the postponement of marriage, not a weakening link between partnership and fertility. We also found some convergence to the patterns of native women among unpartnered African immigrants.

Our findings thus support the socialization hypothesis: we found overwhelming evidence of partnership and family formation patterns among immigrants (especially from South Asia and the Caribbean) that resemble those in their origin countries. Regarding the behaviour of the second generation, their levels of union formation (both marriage and cohabitation) and separation from marriage are in between those of native and immigrant women, providing some evidence for the adaptation hypothesis. However, their family trajectories are often similar to those of immigrants, which supports the minority subculture hypothesis.

We also analysed whether and how the partnership and fertility patterns of immigrants and descendants have changed across birth cohorts. We found the largest change among native unpartnered women: among older cohorts, marriage was the most common outcome, whereas among younger cohorts, cohabitation has become dominant. We did not detect any changes across birth cohorts among different migrant groups. This finding indicates that the interrelationships between the fertility and partnership experiences of immigrants and descendants in the UK have remained stable over time. Among South Asian women, conservative family formation patterns persist even among the youngest cohorts. Similarly, the high risks of separation and births outside unions among Caribbean women are typical across migrant generations and birth cohorts. These findings challenge our expectation that the most recent birth cohorts across all migrant groups and generations would have experienced a significant change in the link between partnership and fertility behaviours (cohort change hypothesis).

These findings also challenge previous research that suggested that the behaviour of the second generation would converge to that of native women in the UK (Berrington 1994, 1996). Although partnership formation is being postponed among the most recent cohort of descendants, the persistence of pathways similar to those of immigrants suggests that their values and preferences remain different from those of native women of the same age. We note that the partnership and fertility histories of the most recent birth cohort are incomplete; thus, it remains for future research to establish whether their partnership and family trajectories will converge to those of the native population. A recent study has shown that the descendants of Indian immigrants have higher expectations of forming a cohabiting union and lower expectations of marrying than women of Pakistani or Bangladeshi origin (Berrington 2020).

The risk of a birth among unpartnered women was higher than expected among South Asian immigrants. Additional analyses (not shown) revealed that the increased birth risk among the unpartnered primarily stemmed from those who had experienced one or more separations rather than from the never partnered. This is in line with previous research that found increasing marital instability and an increasing prevalence of lone parent families among South Asian immigrants (Babb et al. 2006).

We conducted a range of robustness checks. First, we re-estimated the models using information on the time of conceptions rather than births (results not shown) to assess whether premarital/pre-union conceptions were driving some of the results. We found virtually no change in the results except that unpartnered Bangladeshi immigrants were slightly more likely to experience a first conception than a first birth, indicating that ‘shotgun’ marriages might be more common among this group. Second, we distinguished between immigrants who arrived in the UK before age 15 (1.5 generation) and those who arrived as adults (results not shown). The patterns observed among the 1.5 generation were very similar to those of the first generation. Third, the use of retrospective partnership and fertility histories implies that we included information on the experiences of immigrants before arrival in the UK. Replicating the analyses keeping only post-migration episodes for immigrants revealed identical patterns (results not shown).

This study has some limitations. First, there may be some heterogeneity among the second generation which we could not detect. Due to sample size limitations, we were not able to disaggregate the second generation by whether one or both of their parents were immigrants. However, the majority of individuals in the sample had two immigrant parents (except for those with a European/Western background). Similarly, we were unable to analyse the potentially important role of endogamous vs exogamous relationships because information on the origin of previous partners was not available in the retrospective histories. Second, there might be geographical differences in the experiences of immigrants and descendants. For example, the partnership and fertility patterns of those who live in areas where the proportion of ethnic minorities is high may differ from those of women living in areas where it is low. However, this issue could not be studied with the data at hand. Third, there may be unobserved characteristics that jointly influence individuals’ partnership and fertility decisions. Future research should explore these using simultaneous equations models.

Taken together, this study has highlighted that native women as well as European and Western immigrants and descendants are experiencing increasingly diverse family trajectories, with cohabitation, non-marital childbearing, and separation being common experiences. The partnership pathways leading to childbearing among most remaining immigrant and descendant groups have changed less over time. For example, immigrants and descendants from South Asian countries continue to marry first and then have children. Immigrants and descendants of women from the Caribbean region tend to have children outside marriage, form cohabitations, and experience union dissolution. This suggests that the increasing heterogeneity in partnership pathways leading to childbearing is probably here to stay and that British society will remain diverse, with a multitude of partnership and family experiences. Policies aimed at supporting families with children need to account for the heterogeneity of partnership and family trajectories both across individuals’ life courses and across different population subgroups. However, we can still expect to observe gradual changes across migrant generations: fertility is likely to decline further, leading to a reduction in the prevalence of large families, and cohabitation and separation are likely to become more common among most migrant origin groups. These changes may be slower in some minority groups, where marriage continues to be the norm.

## Supplementary Material

Supplementary Material
