# Supplementary material for: Partnership and fertility trajectories of immigrants and descendants in the United Kingdom: A multilevel multistate event history approach
Source: Popul Stud (Camb). 2022 Nov 22;77(3):359–78. doi: 10.1080/00324728.2022.2144639 (PMC10629461; doi:10.1080/00324728.2022.2144639)
Supplement: Supplementary Material [file RPST_A_2144639_SM6697.pdf]

Supplementary material to Partnership and fertility trajectories of immigrants and descendants in the United Kingdom: A multilevel multistate event history approach.

Júlia Mikolai, Hill Kulu. ***Population Studies*****. 2022.**

Table A1 Numbers and proportions of person-months, and partnership and fertility events by categories of variables, women (born 1940–2003) in the UK

Unpartnered women	Outcomes							
	Person-months		Cohabitation		Marriage		Birth	
	N	%	N	%	N	%	N	%
Age								
16–19	1188585	36.25	2968	19.35	3264	26.57	1403	23.03
20–24	849842	25.92	5419	35.32	5461	44.46	1993	32.72
25–29	398296	12.15	3170	20.66	2026	16.49	1349	22.14
30–34	259357	7.91	1673	10.90	683	5.56	789	12.95
35–39	231354	7.05	1034	6.74	396	3.22	431	7.07
40–44	196360	5.99	664	4.33	280	2.28	112	1.84
45–49	155512	4.74	414	2.70	173	1.41	15	0.25
Birth cohort								
1940–59	981944	29.94	2804	18.28	6266	51.01	1312	21.54
1960–79	1623837	49.52	9165	59.74	4870	39.65	3391	55.66
1980–2003	673525	20.54	3373	21.99	1147	9.34	1389	22.80
Time since separation								
0–1 year	115598	3.53	1366	8.90	261	2.12	529	8.68
1–3 years	173112	5.28	1699	11.07	282	2.30	459	7.53
3–5 years	120480	3.67	919	5.99	222	1.81	248	4.07
5+ years	288475	8.80	1467	9.56	445	3.62	373	6.12
Order of separation								
Never partnered	2581642	78.73	9891	64.47	11073	90.15	4483	73.59
Separated once	540061	16.47	4193	27.33	1023	8.33	1336	21.93
Separated two or more times	157604	4.81	1258	8.20	187	1.52	273	4.48
Time since previous birth								
0–1 year	69707	2.13	567	3.70	306	2.49	134	2.20
1–3 years	124391	3.79	737	4.80	267	2.17	1143	18.76
3–5 years	100704	3.07	623	4.06	210	1.71	630	10.34
5+ years	472990	14.42	2178	14.20	720	5.86	768	12.61
Parity								
No child	2511515	76.59	11237	73.24	10780	87.76	3417	56.09
One child	314444	9.59	1956	12.75	763	6.21	1505	24.70
Two or more children	453347	13.82	2149	14.01	740	6.02	1170	19.21
Level of education								
Low	1781767	54.33	6742	43.95	6716	54.68	3973	65.22
Medium	799713	24.39	3948	25.73	2512	20.45	1221	20.04
High	697827	21.28	4652	19.91	3055	44.87	898	14.74
Total	3279306	100	15342	100	12283	100	6092	100

Cohabiting women	Outcomes							
	Person-months		Marriage		Separation		Birth	
	N	%	N	%	N	%	N	%
Age								
16–19	43097	5.98	360	4.78	518	9.31	632	11.61

20–24	180453	25.03	1943	25.82	1701	30.57	1784	32.78
25–29	179357	24.88	2411	32.04	1347	24.20	1527	28.06
30–34	116411	16.15	1330	17.68	828	14.88	887	16.30
35–39	89260	12.38	737	9.80	568	10.21	478	8.78
40–44	66355	9.20	443	5.89	402	7.22	130	2.39
45–49	45926	6.37	300	3.99	201	3.61	4	0.07
Birth cohort								
1940–59	139338	19.33	1876	24.93	664	11.93	594	10.92
1960–79	471261	65.37	4768	63.37	3519	63.23	3585	65.88
1980–2003	110260	15.30	880	11.70	1382	24.83	1263	23.21
Duration of cohabitation								
0–1 year	230059	31.91	2877	38.24	1745	31.36	1608	29.55
1–3 years	182229	25.28	2338	31.07	1756	31.55	1761	32.36
3–5 years	103400	14.34	1070	14.22	820	14.73	888	16.32
5+ years	205171	28.46	1239	16.47	1244	22.35	1185	21.78
Union order								
First union	460257	63.85	4942	65.68	3467	62.30	3829	70.36
Second or higher order union	260602	36.15	2582	34.32	2098	37.70	1613	29.64
Time since previous birth								
0–1 year	60278	8.36	574	7.63	442	7.94	90	1.65
1–3 years	85454	11.85	751	9.98	572	10.28	1209	22.22
3–5 years	50989	7.07	387	5.14	382	6.86	677	12.44
5+ years	165604	22.97	1285	17.08	1071	19.25	731	13.43
Parity								
No child	358535	49.74	4527	60.17	3098	55.67	2735	50.26
One child	149388	20.72	1331	17.69	1148	20.63	1682	30.91
Two or more children	212936	29.54	1666	22.14	1319	23.70	1025	18.83
Level of education								
Low	332117	46.07	3042	40.43	2421	43.50	3052	56.08
Medium	159060	22.07	1705	22.66	1454	26.13	1173	21.55
High	229681	31.86	2777	36.91	1690	30.37	1217	22.36
Total	720859	100	7524	100	5565	100	5442	100

Married women			*Outcomes*			
	Person-months		Separation		Birth	
	N	%	N	%	N	%
Age						
16–19	58683	1.73	140	2.60	1499	5.12
20–24	406510	11.96	851	15.78	7778	26.57
25–29	645004	18.98	1193	22.13	9844	33.63
30–34	663607	19.53	1180	21.88	6731	23.00
35–39	637114	18.75	979	18.16	2889	9.87
40–44	545176	16.04	663	12.30	500	1.71
45–49	442165	13.01	386	7.16	29	0.10
Birth cohort						
1940–59	1826094	53.74	2477	45.94	13537	46.25
1960–79	1471813	43.31	2680	49.70	14020	47.90
1980–2003	100350	2.95	235	4.36	1713	5.85
Duration of marriage						
0–1 year	350798	10.32	695	12.89	4447	15.19
1–3 years	397426	11.69	791	14.67	7589	25.93
3–5 years	357227	10.51	725	13.45	6246	21.34
5+ years	2292807	67.47	3181	58.99	10988	37.54
Union order						
First union	3008737	88.54	4409	81.77	26973	92.15
Second or higher order union	389521	11.46	983	18.23	2333	7.97

Time since previous birth						
0–1 year	339790	10.00	464	8.61	634	2.17
1–3 years	552898	16.27	913	16.93	9232	31.54
3–5 years	356046	10.48	655	12.15	4347	14.85
5+ years	1431906	42.14	2099	38.93	2872	9.81
Parity						
No child	717256	21.11	1261	23.39	12185	41.63
One child	688394	20.26	1170	21.70	10455	35.72
Two or more children	1992608	58.64	2961	54.91	6630	22.65
Level of education						
Low	1814079	53.38	2862	53.07	15415	52.66
Medium	596753	17.56	1133	21.01	5326	18.19
High	987424	29.06	1397	25.91	8529	29.14
Total	3398258	100	5392	100	29270	100

Notes: Unpartnered women refers to never partnered and separated women.
Source: Authors' calculations based on data from the UK Household Longitudinal Study (UKHLS), 2009–19.

Table A2 Outcomes for unpartnered women: relative risks of cohabitation, marriage, and childbirth in the UK

	RR	Sig
Constant	0.01	***
Age		
16–19	0.44	***
20–24 (ref)	1	
25–29	0.97	*
30–34	0.66	***
35–39	0.43	***
40–44	0.29	***
45–49	0.21	***
Birth cohort		
1940–59 (ref)	1	
1960–79	0.95	***
1980–2003	0.79	***
Migrant origin x type of transition		
Native x cohabitation	1.57	***
1G Europe & Western x cohabitation	1.40	***
1G India x cohabitation	0.18	***
1G Pakistan x cohabitation	0.08	***
1G Bangladesh x cohabitation	0.05	***
1G Caribbean x cohabitation	0.74	**
1G Africa x cohabitation	0.58	***
1G Other x cohabitation	0.79	***
2G Europe & Western x cohabitation	1.41	***
2G India x cohabitation	0.54	***
2G Pakistan x cohabitation	0.18	***
2G Bangladesh x cohabitation	0.13	***
2G Caribbean x cohabitation	1.02	
2G Africa x cohabitation	0.82	
2G Other x cohabitation	1.16	**
Native x marriage (ref)	1	
1G Europe & Western x marriage	0.80	***
1G India x marriage	2.11	***
1G Pakistan x marriage	2.52	***
1G Bangladesh x marriage	3.68	***
1G Caribbean x marriage	0.49	***
1G Africa x marriage	0.96	
1G Other x marriage	0.95	
2G Europe & Western x marriage	0.79	***
2G India x marriage	1.39	***
2G Pakistan x marriage	1.93	***
2G Bangladesh x marriage	0.96	
2G Caribbean x marriage	0.23	***
2G Africa x marriage	0.53	***
2G Other x marriage	0.41	***
Native x birth	0.47	***
1G Europe & Western x birth	0.31	***
1G India x birth	0.27	***
1G Pakistan x birth	0.45	***
1G Bangladesh x birth	0.53	**

1G Caribbean x birth	1.34	***
1G Africa x birth	0.71	***
1G Other x birth	0.61	***
2G Europe & Western x birth	0.48	***
2G India x birth	0.28	***
2G Pakistan x birth	0.48	***
2G Bangladesh x birth	0.25	***
2G Caribbean x birth	0.98	
2G Africa x birth	0.45	***
2G Other x birth	0.60	***
Time since separation		
No separation (ref)	1	
0–1 year	1.65	***
1–3 years	1.33	***
3–5 years	1.27	***
5+ years	1.29	***
Order of separation		
Separated less than twice (ref)	1	
Separated two or more times	1.19	***
Time since previous birth		
No birth (ref)	1	
0–1 year	1.21	***
1–3 years	1.35	***
3–5 years	1.17	***
5+ years	1.02	
Parity		
Less than two children (ref)	1	
Two or more children	0.81	***
Level of education		
Low (ref)	1	
Medium	0.86	***
High	1.05	**
ln-L	-67902.3	
N	27943	

Notes: * $p < 0.05$; ** $p < 0.01$; *** $p < 0.001$. Unpartnered women refers to never partnered and separated women. Ref indicates the reference category. RR refers to relative risks.
Source: Authors' calculations based on data from the UK Household Longitudinal Study (UKHLS), 2009–19.

Table A3 Outcomes for cohabiting women: relative risks of marriage, separation, and childbirth in the UK

	RR	Sig
Constant	0.01	***
Age		
16–19	1.17	***
20–24 (ref)	1	
25–29	1.05	*
30–34	0.98	
35–39	0.77	***
40–44	0.57	***
45–49	0.42	***
Birth cohort		
1940–59 (ref)	1	
1960–79	0.96	
1980–2003	1.04	
Migrant origin x type of transition		
Native x marriage	1.43	***
1G Europe & Western x marriage	1.50	***
1G India x marriage	2.64	***
1G Pakistan & Bangladesh x marriage	2.00	*
1G Caribbean x marriage	1.63	**
1G Africa x marriage	1.66	***
1G Other x marriage	1.62	***
2G Europe & Western x marriage	1.35	***
2G India x marriage	1.42	*
2G Pakistan & Bangladesh x marriage	1.84	**
2G Caribbean x marriage	0.75	*
2G Africa x marriage	1.16	
2G Other x marriage	1.12	
Native x separation (ref)	1	
1G Europe & Western x separation	1.26	***
1G India x separation	1.13	
1G Pakistan & Bangladesh x separation	0.67	
1G Caribbean x separation	1.06	
1G Africa x separation	1.18	
1G Other x separation	1.22	**
2G Europe & Western x separation	0.96	
2G India x separation	1.60	**
2G Pakistan & Bangladesh x separation	1.65	*
2G Caribbean x separation	1.43	***
2G Africa x separation	1.64	**
2G Other x separation	1.32	***
Native x birth	1.04	
1G Europe & Western x birth	0.61	***
1G India x birth	1.32	
1G Pakistan & Bangladesh x birth	0.84	
1G Caribbean x birth	1.73	***
1G Africa x birth	1.48	***
1G Other x birth	1.02	
2G Europe & Western x birth	0.96	
2G India x birth	0.70	

2G Pakistan & Bangladesh x birth	1.37	
2G Caribbean x birth	1.44	***
2G Africa x birth	0.92	
2G Other x birth	0.85	
Duration of cohabitation		
0–1 year (ref)	1	
1–3 years	1.12	***
3–5 years	0.96	
5+ years	0.77	***
Union order		
First union (ref)	1	
Second or higher order union	1.14	***
Time since previous birth		
No birth (ref)	1	
0–1 year	0.68	***
1–3 years	1.17	***
3–5 years	1.24	***
5+ years	1.04	
Parity		
Less than two children (ref)	1	
Two or more children	0.80	***
Level of education		
Low (ref)	1	
Medium	0.97	
High	0.93	***
ln-L	-15968.5	
N	11513	

Notes: * p < 0.05; ** p < 0.01; *** p < 0.001. RR refers to relative risks. Ref indicates the reference category.
Source: Authors' calculations based on data from the UK Household Longitudinal Study (UKHLS), 2009–19.

Table A4 Outcomes for married women: relative risks of separation and childbirth in the UK

	RR	Sig
Constant	0.02	***
Age		
16–19	1.41	***
20–24 (ref)	1	
25–29	0.92	***
30–34	0.77	***
35–39	0.46	***
40–44	0.18	***
45–49	0.09	***
Birth cohort		
1940–59 (ref)	1	
1960–79	1.12	***
1980–2003	0.95	*
Migrant origin x type of transition		
Native x separation	0.22	***
1G Europe & Western x separation	0.15	***
1G India x separation	0.05	***
1G Pakistan x separation	0.08	***
1G Bangladesh x separation	0.05	***
1G Caribbean x separation	0.35	***
1G Africa x separation	0.16	***
1G Other x separation	0.15	***
2G Europe & Western x separation	0.26	***
2G India x separation	0.10	***
2G Pakistan x separation	0.18	***
2G Bangladesh x separation	0.11	***
2G Caribbean x separation	0.38	***
2G Africa x separation	0.20	***
2G Other x separation	0.27	***
Native x birth (ref)	1	
1G Europe & Western x birth	1.03	
1G India x birth	1.05	
1G Pakistan x birth	1.55	***
1G Bangladesh x birth	1.32	***
1G Caribbean x birth	1.05	
1G Africa x birth	1.15	***
1G Other x birth	1.13	***
2G Europe & Western x birth	1.03	
2G India x birth	1.13	**
2G Pakistan x birth	1.33	***
2G Bangladesh x birth	1.32	**
2G Caribbean x birth	1.02	
2G Africa x birth	1.14	
2G Other x birth	1.16	**
Duration of marriage		
0–1 year (ref)	1	
1–3 years	1.40	***
3–5 years	1.35	***
5+ years	1.00	
Union order		
First union (ref)	1	

Second or higher order union	1.39	***
Time since previous birth		
No birth (ref)	1	
0–1 year	0.22	***
1–3 years	1.45	***
3–5 years	1.57	***
5+ years	0.86	***
Parity		
Less than two children (ref)	1	
Two or more children	0.45	***
Level of education		
Low (ref)	1	
Medium	0.94	***
High	0.98	
ln-L	-21940.3	
N	17263	

Notes: * p < 0.05; ** p < 0.01; *** p < 0.001. RR refers to relative risks. Ref indicates the reference category.
Source: Authors' calculations based on data from the UK Household Longitudinal Study (UKHLS), 2009–19.
